# Recurrent tertiary hyperparathyroidism due to supernumerary parathyroid glands in a patient receiving long-term hemodialysis: a case report

**DOI:** 10.1186/s12902-019-0346-7

**Published:** 2019-01-28

**Authors:** Tsai-Sung Tai, Yueh-Han Hsu, Jia Ming Chang, Chien-Chin Chen

**Affiliations:** 1 Department of Internal Medicine, Ditmanson Medical Foundation Chia-Yi Christian Hospital, 539 Chung Hsiao Rd, Chiayi City, 600 Taiwan; 2 Department of Medical Research, Ditmanson Medical Foundation Chia-Yi Christian Hospital, 539 Chung Hsiao Rd, Chiayi, 600 Taiwan; 3 Department of Nursing, Min-Hwei Junior College of Health Care Management, 1116, Sec. 2, Zhongshan E. Rd, Liuying Dist, Tainan City, 736 Taiwan; 4 Division of Thoracic Surgery, Department of Surgery, Ditmanson Medical Foundation Chia-Yi Christian Hospital, 539 Chung Hsiao Rd, Chiayi, 600 Taiwan; 5 Department of Pathology, Ditmanson Medical Foundation Chia-Yi Christian Hospital, 539 Chung Hsiao Rd, Chiayi, 600 Taiwan; 6 Department of Cosmetic Science, Chia Nan University of Pharmacy and Science, 60, Sec. 1, Erren Rd, Rende Dist, Tainan, 717 Taiwan

**Keywords:** Hemodialysis, Supernumerary parathyroid glands, Parathyroidectomy, Tertiary hyperparathyroidism, Hungry bone syndrome, Case report

## Abstract

**Background:**

Renal hyperparathyroidism is a common complication of chronic kidney disease (CKD) or end-stage renal disease (ESRD) characterized by elevated parathyroid hormone levels secondary to derangements in the homeostasis of calcium, phosphate, and vitamin D. Rapid correction of severe and prolonged hyperparathyroidism by surgical parathyroidectomy in long-term hemodialysis patients occasionally causes hungry bone syndrome. These patients then exhibit severe and long-lasting secondary or tertiary hyperparathyroidism with high bone turnover.

**Case presentation:**

We report a case of recurrent tertiary hyperparathyroidism after total parathyroidectomy due to supernumerary parathyroid gland in a patient with long-term hemodialysis. Supplementation with intravenous calcium, oral calcium, and vitamin D immediately after patient surgery helps to prevent and treat hungry bone syndrome.

**Conclusions:**

We should prompt a search for the supernumerary parathyroid glands in ESRD patients, who have recurrent or persistent hyperparathyroidism after total parathyroidectomy. ESRD patients are more likely to develop hungry bone syndrome after parathyroidectomy. Prevention and treatment of hungry bone syndrome may be required after ectopic parathyroidectomy in clinical practice.

## Background

Secondary and tertiary hyperparathyroidism occurs commonly in patients with chronic kidney disease (CKD) or end-stage renal disease (ESRD). Previous estimates reported as many as 90% of patients with CKD developed secondary or tertiary hyperparathyroidism by the time they started hemodialysis [1]. Tertiary hyperparathyroidism is a state of autonomously functioning parathyroid tissue typically manifesting as hypercalcemia after either prolonged secondary hyperparathyroidism or successful renal transplantation [2]. Although most of the parathyroid glands are located in eutopic locations, less common ectopic anatomic localization due to variable embryologic migration patterns of the parathyroid glands might occur. Patients with ectopic anatomic localization constitute an etiology of persistent or recurrent hyperparathyroidism after total parathyroidectomy. The incidence of supernumerary parathyroid glands is reported to be between 14.4 and 15% [3, 4]. The most common location of supernumerary parathyroid glands is within the thymus [5].

We report a case of recurrent tertiary hyperparathyroidism after total parathyroidectomy due to supernumerary parathyroid glands in a patient with long-term hemodialysis.

## Case presentation

A 74-year-old Taiwanese man had ESRD secondary to essential hypertension and started hemodialysis therapy since 2002 until now. On 16 June 2005, parathyroid investigations showed the following values: serum intact parathyroid hormone (i-PTH) concentration of 757 pg/ml (reference range 10–73), serum total calcium concentration of 11.2 mg/dl (reference range 8.4–10.2), and serum phosphate concentration of 6.5 mg/dl (reference range 2.7–4.5). As a result, the patient was diagnosed as having tertiary hyperparathyroidism. The ultrasound examination of parathyroid glands revealed the right inferior parathyroid gland 15.5 × 12.0 × 11.9 mm in size and the left inferior parathyroid glands 21.6 × 12.3 × 7.4 mm in size. The patient did not receive the examination of parathyroid scan with Tc-99 m MIBI.

On 5 December 2007, endocrine surgeon performed parathyroidectomy to remove all four parathyroid glands and transplanted right superior parathyroid gland into the subcutaneous fat over the internal part of the right thigh. The pathology of the right and left inferior parathyroid glands showed oxyphil cells and chief cell hyperplasia of both parathyroid tissues. Pre-operative laboratory tests revealed serum i-PTH of 2148 pg/ml, serum total calcium of 11 mg/dl, and serum phosphate of 13.6 mg/dl. Post-operative laboratory tests showed serum i-PTH of 71 pg/ml, serum total calcium of 5.9 mg/dl, and serum phosphate of 8.0 mg/dl.

**Fig. 1:**
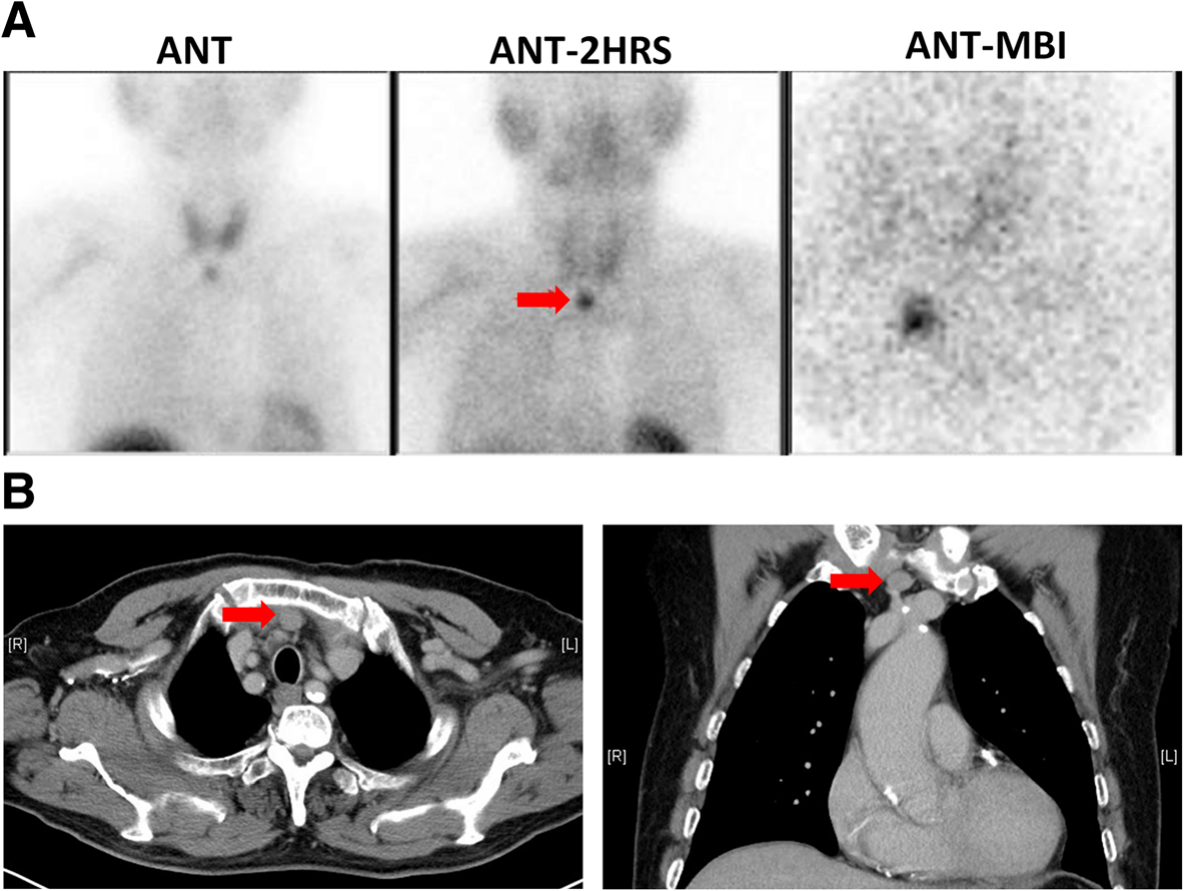
**a** Parathyroid scan with Tc-99 m MIBI, (**b**) Post contrast chest and mediastinal CT scan. The arrow indicates the location of the ectopic parathyroid

In December 2017, the patient was found to have elevated i-PTH concentration again to 1135.9 pg/ml, hypercalcemia (total calcium 11.0 mg/dl) and hyperphosphatemia (phosphate 8.4 mg/dl). Therefore, we performed parathyroid scan with Tc-99 m MIBI and scanned with early and delayed imaging, which showed focal tracer uptake in retrosternal region (Fig. 1A). There was no evidence of recurrent parathyroid gland in the neck or right thigh. Besides, the patient did not have sterna related symptoms or physical findings. So, we suspected ectopic functioning parathyroid gland in the retrosternal region. Post contrast chest and mediastinal computed tomography (CT) scan showed a nodule around 1.3 cm in size in the retrosternal region (Fig. 1B), which can be consistent with an ectopic parathyroid gland. Both investigations revealed evidence of an ectopic parathyroid gland in the retrosternal region.

**Fig. 2:**
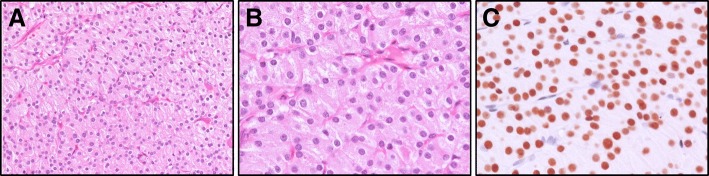
**a** The histopathological examination showed lobules of hyperplastic parathyroid tissues composed primarily of chief cells with thin delicate vascularity, nuclear monomorphism, central round nuclei and granular cytoplasm (200X, H &E stain), (**b**) 400X, H &E stain. **c** Immunohistochemically, the parathyroid tissues are positive for GATA-3, while negative for TTF-1, CD5 and thyroglobulin

On 27 February 2018, a thoracic surgeon performed a neck incision with partial sternotomy and resection of a 1.5 cm mediastinal nodule at the upper mediastinal above the left innominate vein and thymus. The ectopic parathyroid gland is located extra-thymic because we didn’t find any thymic tissue in the histological examination of the resected specimen. Microscopic examination of the specimen showed a parathyroid gland composed of nodular hyperplasia of oxyphil cells and chief cells. Immunohistochemically, the parathyroid gland was positive for GATA-3, while negative for CD5 and synaptophysin (Fig. 2). The pathologic findings were compatible with a diagnosis of an ectopic mediastinal parathyroid gland. We compared the laboratory tests between pre-operation and the second post-operative day: serum i-PTH level decreased from 1135.9 pg/ml to 272.7 pg/ml, serum phosphate level decreased from 7.9 mg/dl to 5.9 mg/dl, and serum total calcium level decreased from 11.0 mg/dl to ionized calcium 0.88 mmol/L (reference range 1.1–1.4). We continued by recording ionized calcium values as 0.81 mmol/L, 0.76 mmol/L, 0.73 mmol/L, 0.95 mmol/L, and 0.85 mmol/L for the past 5 post-operative days, respectively. We immediately administered intravenous calcium chloride 20 ml every 12 h, along with calcium acetate 667 mg four tablets three times a day and vitamin D 0.25 mcg daily from the second post-operative day.

According to the clinical history, the patient was diagnosed to have recurrent tertiary hyperparathyroidism before total parathyroidectomy and ectopic parathyroidectomy. In addition, hungry bone syndrome was present after ectopic parathyroidectomy.

## Discussion and conclusions

Supernumerary (more than four) parathyroid glands may be result from the separation of parathyroid anlage when the glands pull away from the pouch structures during the embryologic branchial complex phase [6]. These additional parathyroid glands are a common cause of recurrent or persistent hyperparathyroidism after parathyroidectomy. Residual small supernumerary glands with diffuse hyperplasia have the potential to be transformed to nodular hyperplasia during long-term hemodialysis [3]. In a retrospective study of 290 patients with renal hyperparathyroidism underwent reoperative parathyroidectomy, supernumerary parathyroid glands were identified in 87 patients (30%), corresponding to intrathymic in 70 cases and to extrathymic in 17 cases [7]. Supernumerary parathyroid glands are thus present in 30% of patients with renal hyperparathyroidism and are also responsible for 32% of persistent or recurrent hyperparathyroidism [7]. Thus, resecting the thymic tongue during the initial operation may reduce the need for reoperative parathyroidectomy to prevent recurrences arising from anterior mediastinal glands [7, 8]. Beside, routine bilateral cervical thymectomy during the initial parathyroidectomy for renal hyperparathyroidism seems to be acceptable and can be recommended in patients on permanent hemodialysis not awaiting kidney transplantation [9, 10].

Tertiary hyperparathyroidism represents an autonomous and advanced form of secondary hyperparathyroidism, which is seen in ESRD patients receiving long-term dialysis and/or kidney transplant. Loss of response to serum calcium concentration leads to parathyroid glands with hyperplasia and autonomous activity, which then leads to elevated serum calcium, phosphate, and PTH levels. When PTH concentration rises, it causes certain complications in bone, vessels or tissues such as osteomalacia, osteoporosis, osteitis fibrosa cystica, vascular calcification, and soft tissue calcification. Operative correction of tertiary hyperparathyroidism is indicated if hypersecretion of PTH and severe or symptomatic hypercalcemia. Parathyroidectomy is considered to be the only curative treatment for patients with tertiary hyperparathyroidism and all parathyroid glands should be examined [11]. In addition, medical treatment of tertiary hyperparathyroidism with Cinacalcet has been reported to be efficacious. A systemic review comparing the outcomes of surgical and medical treatment of tertiary hyperparathyroidism concluded that surgical treatment has higher cure rates than medical therapy [12]. One study reported that the diameter of the parathyroid gland is a main factor involved in resistance to Cinacalcet [13]. Parathyroidectomy for tertiary hyperparathyroidism is associated with lesser rates of renal allograft failure in cinacalcet management [14].

Patients with primary hyperparathyroidism who undergo parathyroidectomy demonstrate a rapid decrease in serum calcium levels after successful removal of one or more hyperactive parathyroid gland(s). Hungry bone syndrome, been coined to the profound (serum calcium < 2.1 mmol/l) and prolonged (longer than 4th day post-operatively) hypocalcaemia, is a condition of hyperdynamic calcium reabsorption into bones following parathyroidectomy [15]. This syndrome manifests as prolonged and symptomatic hypocalcemia. Thus, high doses of calcium, high doses of active metabolites of vitamin D, and adequate correction of magnesium deficiency immediately after surgery helps to treat hypocalcemia. Preoperative treatment with bisphosphonates has been suggested to reduce post-operative hypocalcemia [15]. Preoperative calcitriol therapy can reduce the use of postoperative administration of intravenous calcium by 56% and length of stay by 50% in parathyroidectomy for renal-origin hyperparathyroidism patients [16]. In one study, severe postoperative hypocalcemia requiring intravenous calcium repletion occurred in 97% of ESRD patients with hyperparathyroidism but only 2% of those with primary hyperparathyroidism [17].

In conclusion, ectopic supernumerary parathyroid hyperplasia is an uncommon cause of recurrent tertiary hyperparathyroidism, we should prompt a search for the ectopic parathyroid glands in ESRD patients, who have recurrent or persistent hyperparathyroidism after total parathyroidectomy. ESRD patients are more likely to develop hungry bone syndrome after parathyroidectomy. Prevention and treatment of hungry bone syndrome may be required after ectopic parathyroidectomy in clinical practice.

## Abbreviations

CKD: chronic kidney disease
CT: computed tomography
ESRD: end-stage renal disease
i-PTH: intact parathyroid hormone
